# Naturalistic approach to investigate the neural correlates of a laundry cycle with and without fragrance

**DOI:** 10.1364/BOE.528275

**Published:** 2024-08-23

**Authors:** Giuliano Gaeta, Natalie Gunasekara, Paola Pinti, Andrew Levy, Emilia Parkkinen, Emily Kontaris, Ilias Tachtsidis

**Affiliations:** 1 Health and Well-being Centre of Excellence, Givaudan UK Limited, Ashford, UK; 2 Department of Medical Physics and Biomedical Engineering, University College London, London, UK; 3 Metabolight Ltd, Croydon, UK

## Abstract

Advancements in brain imaging technologies have facilitated the development of “real-world” experimental scenarios. In this study, participants engaged in a household chore – completing a laundry cycle – while their frontal lobe brain activity was monitored using fNIRS. Participants completed this twice using both fragranced and unfragranced detergent, to explore if fNIRS is able to identify any differences in brain activity in response to subtle changes in stimuli. Analysis was conducted using Automatic IDentification of functional Events (AIDE) software and fNIRS correlation-based signal improvement (CBSI). Results indicated that brain activity, particularly in the right frontopolar and occasionally the left dorsolateral prefrontal cortex, was more pronounced and frequent with the unfragranced detergent than the fragranced. This suggests that completing tasks in an environment where a pleasant and relaxing fragrance is present might be less effortful compared to an odourless environment.

## 1. Introduction

Technological advancements over the past thirty years in the neuroimaging field have led to a significant increase in the number and types of devices used in brain imaging research. More researchers are bringing their participants out of the laboratory and into the real world, to study brain responses to specific stimuli and cognitive processes in contexts that are more relevant to the participants themselves [1]. As a result, the development of both hardware systems and research protocols have seen substantial growth in the past decades [2].

At the hardware level, the scene in recent years has been dominated by electroencephalography (EEG) devices [3,4]. New mobile systems can sometimes offer a good trade-off between mobility needs for the participants and accuracy/richness of the data for the experimenter, with some devices reaching as many as 64 channels. Moreover, the wireless communication they offer has allowed researchers to use mobile EEG devices in very different contexts, from the development of brain-computer interfaces (BCIs) [5,6], to neurofeedback protocols [7,8,9].

Very recently, other technologies that were once considered impossible to be used in a mobile setting, like magnetoencephalography (MEG) have also seen the development of prototypes allowing participants to move freely in real-life scenarios [10,11]. However, both EEG and MEG are subject to limitations that impose sometimes strong trade-offs to researchers in order to collect data from participants outside of the lab.

Specifically, EEG is prone to electrical noise from the muscles around the electrodes (facial muscles, tongue, jaw, eye movements) that can severely compromise the quality of the recordings. In fact, brain and muscle signals are both of the electric type, but muscle signals tend to be at least one order of magnitude larger in amplitude [12,13]. Moreover, the already low spatial resolution offered by EEG is further reduced by mobile systems that normally provide very few (<20) electrodes in order to increase comfort for the participants. Additionally, commercially available devices often offer very low sampling rate compared to lab-based devices, therefore not allowing researchers to obtain clear, dense data to analyse and interpret. This is especially important in contexts where data quality is essential, for example when developing new brain markers or investigating new brain signatures through novel paradigms [14]. For MEG, the biggest drawback is that the environment in which both the devices and participants operate needs to be magnetically fully isolated, and mobility for the participants is still limited given the current technologies available [15].

Nowadays, the brain imaging technique that offers the best trade-off between accuracy and mobility is functional near-infrared spectroscopy (fNIRS) [16,17]. fNIRS has been successfully exploited in the past years as a fully mobile technology and this, in turn, has led to the development of a wide range of devices that can be used in real-life environments [18,19,20]. This achievement was, in part, made possible by the unique ability of fNIRS to be easily integrated (both in terms of hardware and data collection) with EEG in mobile systems [21,22].

The continuous cycle of improvements in hardware and equipment leading in turn to the development of novel out-of-the-lab protocols has also facilitated the improvement of data analysis platforms, and the standardisation of best practices for the use and handling of fNIRS data, as well as enabling the creation of tools to further explore fNIRS data [23,24,25]. Among these, the AIDE (Automatic IDentification of functional Events) platform has gained interest in the fNIRS community for its ability to mark significant brain activation events in conditions where the rigorous epoching of the data becomes difficult to obtain due to the nature of the naturalistic protocols [26]. AIDE reverses the traditional approach of analysing brain responses in response to a specific protocol (for example, “what are the brain patterns in response to a participant looking at a picture?”) and instead attempts to determine significant events starting from the analysis of brain activity (i.e. “based on this fNIRS dataset, when is my participant looking at the picture?”). The advantages of this approach should be immediately clear: using tools like AIDE, researchers are able to leave the lab and truly exploit fNIRS in naturalistic settings, where determining a clear sequence of marked events is not always possible [27].

Despite its flexibility and ability to operate in extremely different research contexts, to date, fNIRS has not been widely exploited in the fragrance industry, or more generally, in olfactory research [28]. Moreover, the only significant contributions in the field have been made either in clinical settings [29,30], or in lab-based settings [31]. However, the sense of smell is involved in an extremely wide range of commonplace daily activities: from the scent of a shampoo or conditioner during the morning shower to the candle lit during the yoga session in the evening, we are constantly exposed to odours and actively seek olfactory experience from the consumer products we use. Targeting one such real-life scenario, we assess the feasibility of using fNIRS record brain activity from consumers and provide useful information on the brain processes behind such activities. We chose to use a laundry cycle in our experiment for numerous reasons: it is a truly out-of-the-lab activity that most households perform; it involves a high level of movement and is familiar enough to standard consumers to allow for full replicability of the laundry process without compromising the naturalistic aspect of the test (i.e. without providing too stringent instructions to the participants). Moreover, to investigate the sensitivity of the brain imaging tool, we asked our participants to complete the experiment twice, once with a fragranced detergent and once with an unfragranced detergent, to check if any perceivable differences between the two conditions can be highlighted by fNIRS recordings, with particular interest shown to the prefrontal cortex (PFC), as it is commonly targeted in odour based fNIRS studies.

The aim of the current paper is therefore to investigate whether neuroimaging techniques -specifically fNIRS- can be employed in naturalistic scenarios to study the effects of olfactive stimuli on brain activation patterns in the PFC. To the best of our knowledge, this is the very first example of an fNIRS-based protocol run in the fragrances and flavours industry, where naïve consumers are placed in an ecologically valid environment to perform a test mimicking a real-life scenario, such as doing laundry. The use of fNIRS in combination with AIDE may prove beneficial for studies involving odour, a highly evocative stimulus, where emotional and well-being aspects of human nature are more difficult to both encourage and measure in restrictive, time-controlled environments. We aim to test the efficacy and sensitivity of this paradigm, and provide an example of a data handling pipeline to explore consumer responses to real-life scenarios.

We hypothesise that fNIRS is a suitable tool to record brain activity in a highly noisy context such as a laundry cycle. To prove this point, we expect to find significant differences, in terms of brain activation, between two very similar scenarios in the experiment, where the only difference is the presence or absence of a fragrance in the washing detergent.

## 2. Materials and methods

### 2.1 Participants

16 participants were included in the analysis for this study (age M = 50.9 and SD = 14.7, 5 males and 11 females). The study was approved by Givaudan’s Internal Review Board, ethics no. 2019-002 (equivalent to an ethics committee) and all participants signed the informed consent prior to taking part in the experiment.

### 2.2 Procedure and paradigm

**Fig. 1.**
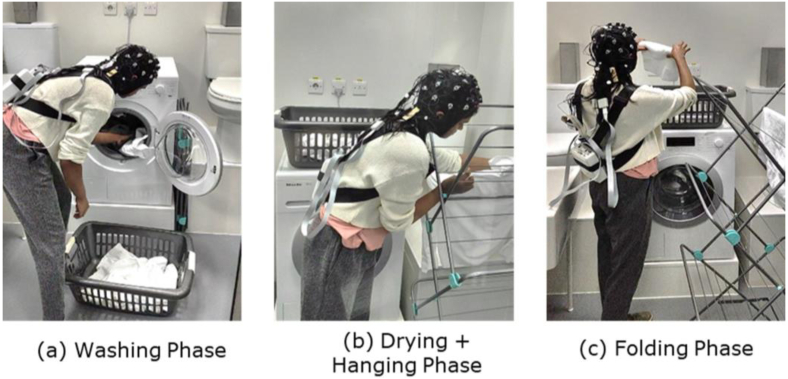
Picture of the three stages of the dynamic phase of the experiment; (a) washing, (b) drying/hanging, (c) folding.

**Fig. 2.**
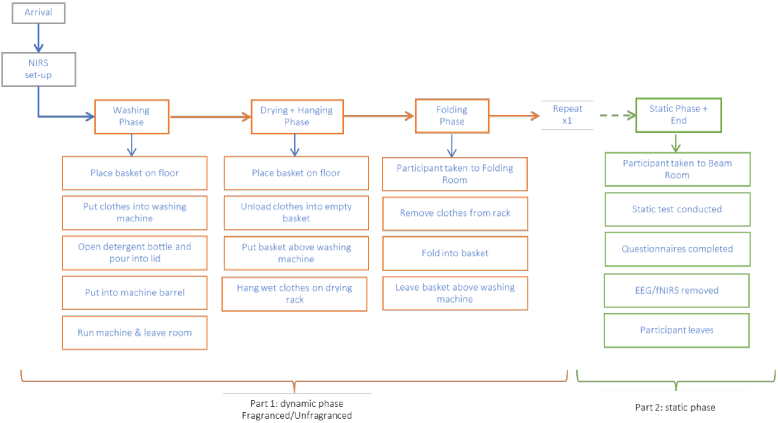
Timeline of the experimental protocol. After the neuroimaging setup, participants carried out the dynamic phase twice followed by the static experiment.

The study was conducted in the laundry booths facilities at the Givaudan site in Ashford, UK. Each of the booths used (10 m^3^, 21 °C, 50% RH (relative humidity)) is equipped with a European front-loading washing machine (Miele Front Loaders WKB120) with a front tray for detergent and fabric conditioner (Fig. 1). For the current experiment, a standard programme was selected before the arrival of the participants, so that they were asked to only press the “Start” button and avoid any confusion or discrepancy across participants during the test. Three booths were used by each participant for the test, and in each booth they performed one of the three phases of the dynamic phase (see details below – Fig. 2). Moreover, the Fragranced and Unfragranced conditions were performed in two different sets of booths to prevent any odour contamination of the environment or the fabrics, particularly due to fabric detergent build-up or carry-over effects of the fragrance.

The entire experimental session consisted of two main parts: 1) the dynamic phase; 2) the static experiment (Fig. 2). All participants first completed the dynamic phase, followed by the static phase. The timeline of the study is show in Fig. 2.

#### 2.2.1 Dynamic experiment

The dynamic phase comprised three main tasks (Fig. 2):

1) Wash: participants were brought into the first booth equipped with a washing machine and a basket of dry clothes placed on top of the washing machine. Each participant received a standard set of laundry to wash, representative of a standard washing machine load. The set contained a total of 1.6 kg of fabric items, comprising shirts, towels and pillow cases. This standard laundry load was used throughout the experiment, including the following two phases. The participants were given instructions to place the basket on the floor, empty the content of the basket into the washing machine, open the plain bottle of detergent and pour it into the bottle’s lid to reach a specified quantity of 20 ml, place it in the washing machine drum and finally start the washing cycle of the washing machine (express 40 °C wash cycle, 1,000 spin/min). No specific instruction was given on how to handle the detergent (for example, if smelling it before pouring it in the cap) in order to maintain the highest level of naturalistic setting possible. At this point, they were asked to leave the booth.
2) Dry + Hang: participants were brought into the second booth equipped with pre-washed laundry inside the washing machine. The washing cycle had ended between 30 and 45 minutes before the participant started this phase, with the aim of providing the same experience to all the participants in terms of fragrance effects (e.g. the blooming effect once opening the washing machine door) and other sensorial aspects (e.g. the level of dampness of the wet clothes or the temperature of the clothes after the washing cycle). Participants were instructed to open the washing machine’s door, empty the content into a basket, and hang the clothes onto a standard three-tier v-shaped airer one at a time. Upon completion, they were now asked to move to the next booth.
3) Fold: participants were brought into the third booth equipped with an airer holding dried clothes. These clothes (the standard set) were previously washed in the respective detergent and line dried the night prior to the test. All the sets of items were washed 24 hours before the use by each participant, therefore ensuring that all the participants had the same experience while interacting with the dried load. In this phase, participants were asked to remove the clothes from the rack and fold them into a basket and leave the basket on top of the washing machine.

The dynamic phase was performed twice by each participant, once with a fragranced detergent (Surf Tropical Lily & Ylang Ylang) and once with an unfragranced detergent (Surcare liquid washing up). The order of the Fragranced / Unfragranced conditions was counterbalanced across participants.

#### 2.2.2 Static experiment

The static experiment was conducted in a different room, separate from the booths. Participants were asked to smell the detergents in three different forms: on dry cloth after washing the cloth with the detergent and air-drying it; on wet cloth, immediately after washing the cloth with the detergent; or as the original liquid detergent. Detergents were either fragranced or unfragranced, and participants completed a total of six smelling trials. The order of the detergent forms and Fragranced / Unfragranced conditions was counterbalanced across participants. Immediately after smelling each sample, participants completed a questionnaire, rating each item on 5 attributes: liking (9-point scale from ‘I dislike it extremely’ to ‘I like it extremely’); intensity (9-point scale from ‘extremely weak’ to ‘extremely strong’); invigorating (9-point scale from ‘not invigorated at all’ to ‘extremely invigorated’); relaxing (9-point scale from ‘not relaxed at all’ to ‘extremely relaxed) and just about right (5-point scale from ‘much too weak for me’ to ‘much too strong for me’).

### 2.3 fNIRS data acquisition

**Fig. 3.**
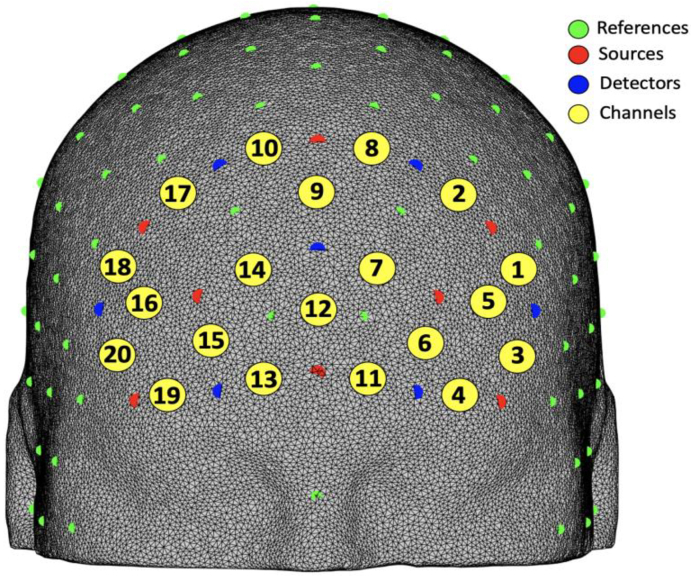
fNIRS channel configuration. The 10-10 EEG electrode placement systems references are shown in green; light sources and detectors are represented by red and blue dots respectively; measurement channels are shown in yellow.

Changes in oxygenated (HbO_2_) and deoxygenated (HbR) haemoglobin over the prefrontal cortex was recorded using a mobile fNIRS system (NIRSPort2, NIRx Medical Technologies LLC, Berlin, Germany) using a standard NIRx PFC optode configuration (Fig. 3). The fNIRS system covered the prefrontal and frontal areas of the brain. The fNIRS device consisted of eight light sources (λ_1_= 760 nm; λ_2_= 850 nm) and seven detectors were arranged at a relative distance of 3 cm, creating 20 measurement channels. The anatomical locations for each channel can be viewed in Table 1, found in the appendices . These were computed using the anatomical co-registration method implemented in the NIRS-SPM software package; the percentage of overlap of each channel onto anatomical regions was estimated calculating the overlap of a 10 mm radius sphere centred around the cortically-projected coordinate of the channels [32].

### 2.4 fNIRS data analysis

Raw intensity signals’ quality was assessed both visually and with the QT-NIRS software package (https://github.com/lpollonini/qt-nirs) [33] to identify signals with poor quality due to e.g., poor optical coupling, saturation, or too many motion artifacts. The Scalp Contact Index (SCI) and Peak Spectral Power (PSP) were computed in non-overlapping time windows of 5 seconds to include at least 4-5 harmonics of the fundamental heart rate frequency (∼1 Hz); channels that had an SCI < 0.6 and PSP < 0.1 in more than 50% of the time windows were marked as noisy and excluded from further analyses. For participant number 22 (P22), channel 4 and 6 were further excluded following visual inspection due to a large number of cap decoupling’s in the second half of the experiment. Two participants were excluded completely as all their fNIRS signals were marked as noisy.

Data from the included channels were pre-processed using the Homer2 software package [34]. Raw intensity signals were first converted into changes in optical density (function: *hmrIntensity2OD*); motion artifacts were then corrected using the wavelet-based method (function: *hmrMotionCorrectWavelet*; *iqr = 1.5*; [35], and low and high frequency noise were reduced using a 5^th^ order Butterworth band-pass filter (function: *hmrBandPassFilt; [0.01 0.3] Hz*). Changes in HbO_2_ and HbR were finally computed through the modified Beer Lambert law considering a DPF of 6 (function: *hmrOD2Conc; DPF = *[6 6]).

Given the naturalistic structure of the task, fNIRS data were analysed using the Automatic IDentification of functional Events (AIDE) method [26]. The pre-processed HbO_2_ and HbR signals were first combined into the activation signals through the correlation-based signal improvement algorithm (CBSI) [36], further helping in accounting for systemic interferences [37]. The activation signals from the 20 channels were down-sampled to 1 Hz and entered into AIDE. These were fitted with a canonical response function convolved with boxcar functions reflecting all possible event onset and durations (up to 230 s long) to identify the occurrence of functional events. For each participant and channel, each functional event output includes information about the onset and duration of functional activation, and the t-value (representing the goodness of fit of the General Linear Model and hence the strength of activation).

The detected functional events were assigned to each experimental condition (Dynamic: Fragranced/Unfragranced wash, dry + hang, fold; Static: Fragranced/Unfragranced liquid, wet, dry) by finding the condition each identified onset was closest to. We then extracted the following parameters that reflect various aspects of brain activity:

• The average number of detected events (Mean_ONSET_), representing the average frequency of brain activity (how many functional events occur);
• The average duration of the events (Mean_DUR_), representing the average duration of brain activity;
• The average t-values (Mean_t-VALUE_), representing the average strength of brain activity;
• The maximum t-values (Max_t-VALUE_), representing the maximum strength of brain activity (peak brain activity).

These were computed across all the events identified for each channel and condition of each participant. The abovementioned parameters (Mean_ONSET_, Mean_DUR_, Mean_t-VALUE_, Max_t-VALUE_) were entered into channel-wise paired sample t-tests to test if there are any significant differences (p < 0.05) in the patterns of brain activity when actions are performed in the presence of fragranced and unfragranced detergents. In particular, we tested the hypothesis that the fragranced detergent led to significantly more frequent, longer and stronger brain activations than the unfragranced detergent, comparing the following conditions:

1) Dynamic Fragranced wash > Dynamic Unfragranced wash
2) Dynamic Fragranced dry + hang > Dynamic Unfragranced dry + hang
3) Dynamic Fragranced fold > Dynamic Unfragranced fold
4) Static Fragranced dry > Static Unfragranced dry
5) Static Fragranced liquid > Static Unfragranced liquid
6) Static Fragranced wet > Static Unfragranced wet

In order to take into account the varying durations of the experimental conditions across participants, the same comparisons (1-6) were also carried out on Mean_ONSET_, Mean_DUR_, Mean_t-VALUE_, Max_t-VALUE_ normalized by each participant’s duration of the corresponding condition (Fragranced/Unfragranced wash, dry + hang, fold). The normality of the distributions was assessed using the Kolmogorov-Smirnov test at 0.05 significance. Results were corrected for multiple comparisons using the False Discovery Rate (FDR) at q < 0.05 [38].

#### 2.4.1 Correlation between fNIRS data and fragrance questionnaire

In order to assess the presence of correlations between the brain activation patterns and behavioural responses to fragrances, the Person’s correlation coefficients r between Mean_ONSET_, Mean_DUR_, Mean_t-VALUE_, Max_t-VALUE_ and the questionnaire scores for each attribute (liking, intensity, invigorating, relaxing, just about right) were computed for each channel and condition of the dynamic phase. In particular, Mean_ONSET_, Mean_DUR_, Mean_t-VALUE_, Max_t-VALUE_ from the dynamic wash condition were correlated with the questionnaire scores for the liquid detergent; Mean_ONSET_, Mean_DUR_, Mean_t-VALUE_, Max_t-VALUE_ from the dynamic hang & dry condition were correlated with the questionnaire scores for the wet detergent; Mean_ONSET_, Mean_DUR_, Mean_t-VALUE_, Max_t-VALUE_ from the dynamic dry condition were correlated with the questionnaire scores for the dry detergent.

## 3. Results

**Fig. 4.**
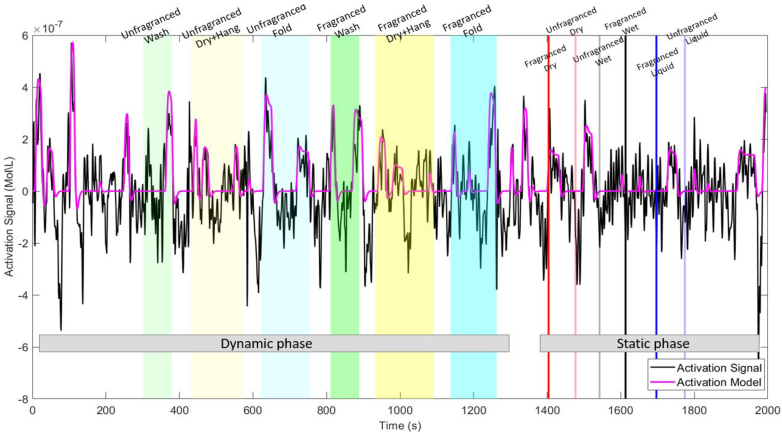
Example activation model determined by AIDE (magenta line) corresponding to the best fit between the activation signal (black line) and the convolution of the canonical HRF and the boxcar function with the optimal onset and duration. Shaded coloured areas represent the various experimental conditions. The example refers to channel 2 of P24.

The AIDE analysis identified when functional activation occurred within each condition, determining the onset and duration as well as the strength of activation for each channel. This was based on the best fit obtained through the convolution of the canonical HRF and a boxcar function with the onsets and durations. An example of the resulting activation model is shown in Fig. 4 for one channel of one participant (Channel 2, P24). Shaded coloured areas represent the various conditions; the CBSI activation signal for channel 2 is represented in black and the activation model (i.e. the best fit) is shown in magenta.

**Fig. 5.**
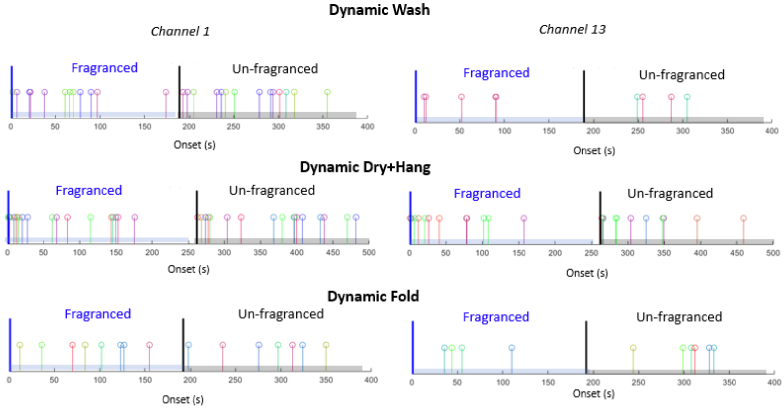
Example of timeline of the onsets of functional events detected by AIDE for two representative channels for the Dynamic Wash, Dry + Hang, Fold conditions. Onsets for each participant are shown with a different colour.

An example of the output is presented in Fig. 5. For this example, we selected two channels, 1 and 13. The figure shows the timeline of the onsets of all functional events for all participants in these two channels, for both the Fragranced and Unfragranced dynamic phase conditions. Each vertical line represents the identified onset of the functional activity, while the various colours correspond to the different participants. See appendices for the remaining channel information.

Highly detailed results of the statistical analyses are reported in the Appendix.

### 3.1 Initial results

For the Dynamic phase, channel-wise paired sample t-tests were applied to Mean_ONSET_, Mean_DUR_, Mean_t-VALUE_, Max_t-VALUE_ to test whether the use of fragranced detergents led to significant differences (p < 0.05) in the patterns of brain activity at the group level compared to unfragranced detergents when people were washing, hanging or folding clothes.

**Fig. 6.**
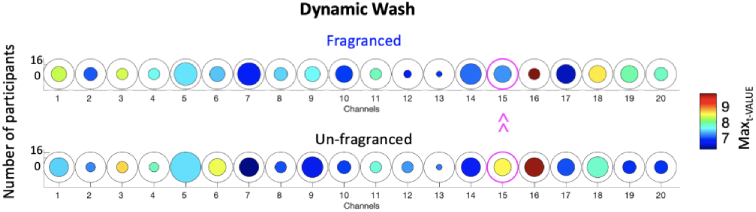
Group-level results for the paired sample t-test comparing the Max_t-VALUE_ of the Fragranced condition to the Unfragranced condition for the dynamic wash phase. Circles represent individual channels and the colour bar indicates the average group Max_t-VALUE_. The channels circled in magenta represents those with a significant difference between Fragranced and Unfragranced conditions (p < 0.05).

For the dynamic wash condition, the Max_t-VALUE_ was significantly higher in the Unfragranced condition compared to the Fragranced condition in Channel 15 (*t(5)*=-3.80, p < 0.05; not surviving FDR correction) corresponding to higher peak activity. No significant differences (p > 0.05) were found in terms of Mean_ONSET_, Mean_DUR_, Mean_t-VALUE_. In Fig. 6, it is shown the group-averaged Max_t-VALUE_ for each channel of the Fragranced wash condition (top) and Unfragranced wash condition (bottom). The colour bar represents the Max_t-VALUE_ while the radius of the circle represents the number of participants that presented a functional event of each channel and condition across the full sample of 16 individuals. The channels circled in magenta represent those with a significant difference between the Fragranced and Unfragranced conditions (p < 0.05).

**Fig. 7.**
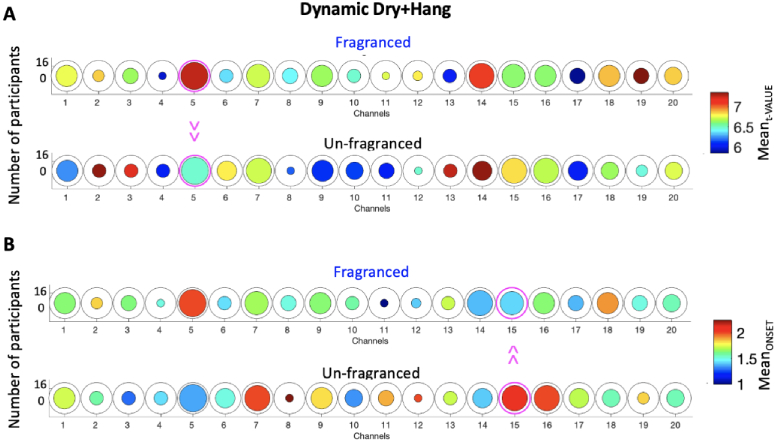
Group-level results for the paired sample t-test comparing the Mean_t-VALUE_ (A) and Mean_ONSET_ (B) of the Fragranced condition to the Unfragranced condition for the dry + hang phase. Circles represent individual channels and the colour bar indicate the average group Mean_t-VALUE_/Mean_ONSET_. The channels circled in magenta represent those with a significant difference between Fragranced and Unfragranced conditions (p < 0.05).

For the dynamic dry + hang condition, the Mean_t-VALUE_ was significantly higher in the Fragranced condition compared to the Unfragranced condition in Channel 5 (*t(12) *= 2.31, p < 0.05; not surviving FDR correction) corresponding to a higher average level of brain activity (Fig. 7(A)). There was more frequent brain activity in the Unfragranced condition in Channel 15 compared to the Fragranced condition (Fig. 7(B)) as revealed by a significantly higher Mean_ONSET_ (*t(10)*=-2.63, p < 0.05; not surviving FDR correction). No significant differences (p > 0.05) were found in terms of Mean_DUR_ and Max_t-VALUE_.

**Fig. 8.**
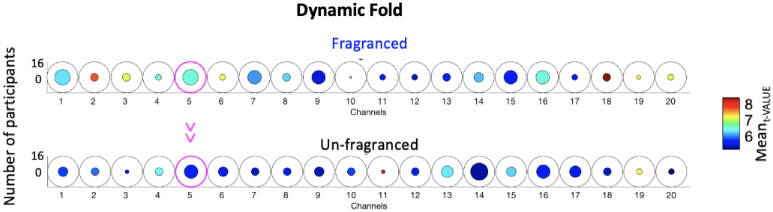
Group-level results for the paired sample t-test comparing the Mean_t-VALUE_ of the Fragranced condition to the Unfragranced condition for the dynamic fold phase. Circles represent individual channels and the colour bar indicate the average group Mean_t-VALUE_. The channels circled in magenta represent those with a significant difference between Fragranced and Unfragranced conditions (p < 0.05).

Similarly, for the dynamic fold condition, the Mean_t-VALUE_ was significantly higher in the Fragranced condition compared to the Unfragranced condition in Channel 5 (*t(4) *= 3.63, p < 0.05; not surviving FDR correction) corresponding to a higher average level of brain activity (Fig. 8); no significant differences (p > 0.05) were found in terms of Mean_ONSET_, Mean_DUR_, Mean_t-VALUE_.

For the static experiment, channel-wise paired sample t-tests were applied to Mean_ONSET_, Mean_DUR_, Mean_t-VALUE_, Max_t-VALUE_ to test whether smelling dry, wet or liquid fragranced detergents led to differences in the patterns of brain activity at the group level respect to smelling dry, wet or liquid unfragranced detergents.

**Fig. 9.**
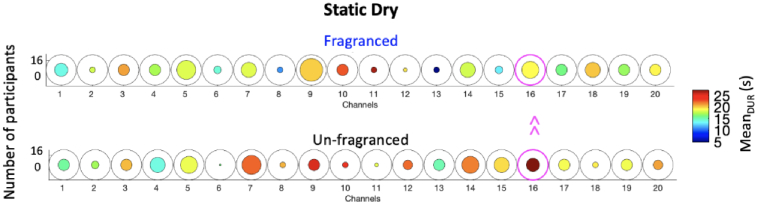
Group-level results for the paired sample t-test comparing the Mean_DUR_ of the Fragranced condition to the Unfragranced condition for the Static dry condition. Circles represent individual channels and the colour bar indicate the average group Mean_DUR_. The channels circled in magenta represent those with a significant difference between Fragranced and Unfragranced conditions (p < 0.05).

For the static dry condition, smelling unfragranced dry detergent led to longer functional activation events (Fig. 9) as revealed by significantly higher Mean_DUR_ in the Unfragranced condition compared to the Fragranced condition in Channel 16 (*t(5)*=-3.80, p < 0.05; not surviving FDR correction). No other significant differences (p > 0.05) were found.

No significant differences were found for the static wet and liquid conditions.

### 3.2 Results on normalised data

To account for the different duration of the dynamic phasal conditions (Fragranced/Unfragranced wash, dry + hang, fold) across participants, channel-wise paired sample t-tests were applied to Mean_ONSET_, Mean_DUR_, Mean_t-VALUE_, Max_t-VALUE_ normalised by the duration of the corresponding condition of each participant.

**Fig. 10.**
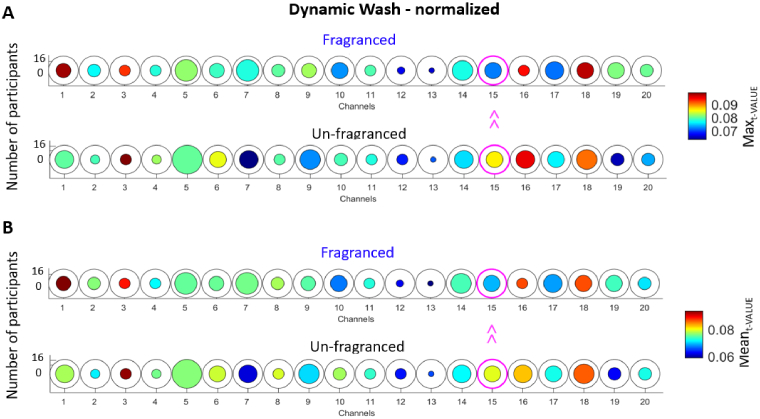
Group-level results for the paired sample t-test comparing the Max_t-VALUE_ (A) and Mean_t-VALUE_ (B) of the Fragranced condition to the Unfragranced condition for the wash phase. Circles represent individual channels and the colour bar indicate the average group Max_t-VALUE_/ Mean_t-VALUE_. The channels circled in magenta represent those with a significant difference between Fragranced and Unfragranced conditions (p < 0.05).

In this case, in the washing condition the normalised Max_t-VALUE_ was significantly higher in the Unfragranced condition compared to the Fragranced condition in Channel 15 (*t(5)*=-3.75, p < 0.05; not surviving FDR correction) corresponding to higher peak activity. Moreover, the normalised Mean_t-VALUE_ was significantly higher in the Unfragranced condition compared to the Fragranced condition in Channel 15 (*t(5)*=-2.66, p < 0.05; not surviving FDR correction) corresponding to a higher average level of brain activity (Fig. 10); no significant differences (p > 0.05) were found in terms of normalized Mean_ONSET_ and Mean_DUR_.

**Fig. 11.**
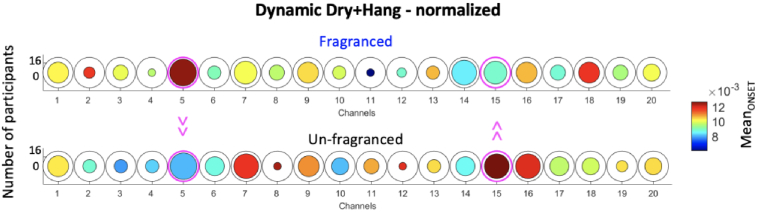
Group-level results for the paired sample t-test comparing the Mean_ONSET_ of the Fragranced condition to the Unfragranced condition for the dynamic dry + hang condition. Circles represent individual channels and the colour bar indicate the average group Mean_ONSET_. The channels circled in magenta represent those with a significant difference between Fragranced and Unfragranced conditions (p < 0.05).

For the dynamic dry + hang condition, there was more frequent brain activity in the Unfragranced condition in Channel 15 compared to the Fragranced condition (Fig. 11) as revealed by a significantly higher normalised Mean_ONSET_ (*t(10)*=-2.33, p < 0.05; not surviving FDR correction), and more frequent brain activity in the Fragranced condition respect to the Unfragranced condition in Channel 5 (*t(12) *= 2.49, p < 0.05; not surviving FDR correction). No significant differences (p > 0.05) were found in terms of normalised Mean_DUR,_ Mean_t-VALUE,_ and Max_t-VALUE_.

### 3.3 Correlation results between fNIRS data and questionnaire

The significant results for the correlation analysis are summarised in Table 1.

**Table 1.**
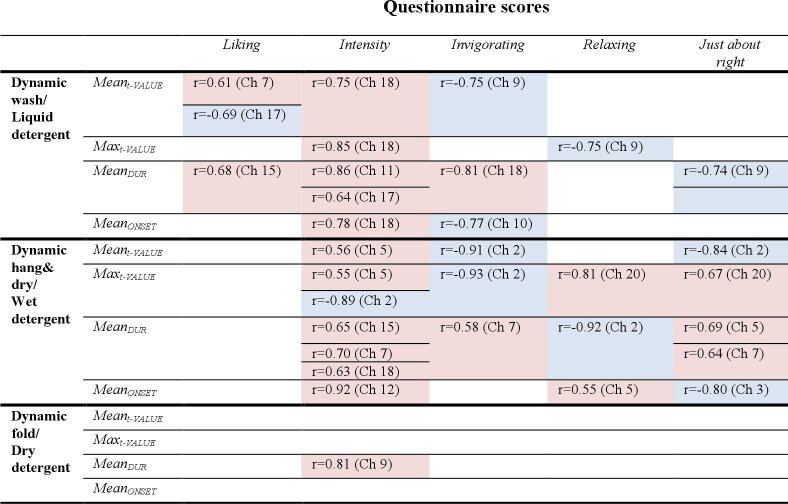
Summary of the significant correlations between the AIDE-derived parameters and the scores from the questionnaires. Pearson’s correlation coefficients r are reported as well as the channels where the correlation was significant. Red cells indicate positive correlations; blue cells indicate negative correlations.

Overall, the first two stages of the dynamic phase showed the highest number of correlations between AIDE parameters and questionnaires scores. In the final stage, the fold, there was only one significant correlation between the Mean_DUR_ scores in channel 9 and the intensity scores.

Intensity scores positively correlated with most of the AIDE parameters in the first two stages. During the wash part, with Max_t-VALUE_, Mean_ONSET_ and Mean_t-VALUE_ in channel 18, with Mean_DUR_ in channels 11 and 17; during the wash part with Max_t-VALUE_ and Mean_t-VALUE_ in channel 5, with Mean_DUR_ channels 7, 15 and 18 and with Mean_ONSET_ in channel 12. In this second part, the only negative correlation was with Max_t-VALUE_ in channel 2.

Liking scores correlated with AIDE parameters only in the wash stage: we observed positive correlations with Mean_t-VALUE_ (channel 7) and Mean_DUR_ (channel 15), and negative correlations with Mean_t-VALUE_ (channel 17).

Invigorating scores positively correlated with Mean_DUR_ in both the wash stage (channel 18) and the hang stage (channel 7), while negative correlations were observed in the wash stage with Mean_t-VALUE_ (channel 9) and Mean_ONSET_ (channel 10), and in the hang stage with Mean_t-VALUE_ and Max_t-VALUE_ (both in channel 2).

In the wash stage, we observed negative correlations between Max_t-VALUE_ and relaxing scores, and between Mean_DUR_ and “just about right” scores (both in channel 9), while in the hang stage, we observed positive correlations between Max_t-VALUE_ and both relaxing and “just about right” scores (channel 20), other than positive correlations between the “just about right” scores and Mean_DUR_ (channels 5 and 7), and between relaxing scores and Mean_ONSET_ in channel 5. Finally, there was a negative correlation between Mean_DUR_ and relaxing scores (channel 2) and between “just about right” scores and both Mean_t-VALUE_ (channel 2) and. Mean_ONSET_ (channel 3) again in the hang stage.

## 4. Discussion

The constant improvement of mobile brain imaging technologies has allowed the parallel development of many out-of-the-lab protocols in brain imaging research to investigate brain patterns when participants are immersed in more naturalistic environments. However, to date, no such approach has been considered in the olfactory research world. This study aimed to investigate whether neuroimaging techniques -specifically fNIRS- can be employed in naturalistic scenarios to study the effects of olfactive stimuli on brain activation patterns in the PFC.

We employed a protocol reproducing the main three steps of a laundry cycle: putting the clothes in the washing machine and starting the washing cycle; emptying the washing machine and putting the wet clothes on an airer; taking the dry clothes from the airer and folding them. Using real consumer products, we collected fNIRS data from our participants while they completed the cycle twice, with a fragranced and an unfragranced fabric detergent (dynamic phase of the study). After, participants explicitly rated the liquid detergents, the wet cloths and the dry cloths on a series of 9-points attribute scales (static phase). Our results suggest that wearable fNIRS can be a valuable tool in olfactory research and that different patterns of activity can be observed in the PFC in response to olfactive stimuli and during different phases of a realistic washing cycle.

Overall, results indicate that the unfragranced detergents lead to more frequent and stronger brain activity than conditions with fragranced detergents, both in the dynamic and static phases (with the exception of a few instances where Fragranced > Unfragranced). Most of the time, this was localised in Channel 15 (right frontopolar PFC) and sometimes in Channel 5 (left dorsolateral PFC). The mere presence of differences across two very similar conditions is inherent evidence of the ability of fNIRS to highlight changes in brain activity exclusively due to the olfactive properties of the stimuli. We chose to benchmark our fragranced condition against an odourless benchmark rather than a malodour, in which case the differences might have been more evident [39]. This difference can be interpreted in terms of “higher cost” to perform the actions in the environment in the absence of a fragrance, compared to when a pleasant fragrance is present [40]. Therefore, participants appear to find the same actions less effortful if a pleasant fragrance is present, possibly due to a sense of relaxation given by the fragrance itself [41,42]. This hypothesis is corroborated by a significantly different level of activity in the frontopolar PFC, located in Brodmann’s area 10. Previous research has highlighted how less pleasant experimental conditions activate this to a greater extent than more pleasant conditions, even when the stimulation involves the chemical senses [43]. With regards to the dorsolateral PFC, located in Brodmann’s area 9, it has often been found active in studies investigating olfactive stimuli, particularly in the left hemisphere [44], but also in protocols involving moderate physical activity, as in our study, where possibly the Unfragranced condition proves to be more challenging for the participants [45]. The activation of this area has also been associated with less pleasant stimulation compared to a more pleasant one, as the absence vs. presence of a fragrance in our study suggests [46] and, more generally, to emotional regulation, as could be the case when processing hedonic features of a fragranced (or unfragranced) product [47]. It is also worth noting how several regions of the prefrontal cortex are both anatomically and functionally connected to both emotional-relevant centres in the limbic system and to olfaction-related areas [48]. Thus, the aforementioned activations can simply be the result of the processing of olfactive stimuli, or the difficulty associated with the processing, in the case of the unfragranced detergent.

In terms of correlation between explicit ratings and brain activity in the static phase of our study, it seems that olfactive stimuli that were rated as more intense were associated with stronger, more frequent, and longer brain activity. This is consistent with previous research showing how brain activity in response to fragrances increases with the increase of the strength of the odour [49,50]. It is worth mentioning that this is the first time such a measurement has been carried out using fNIRS, since previous research only employed EEG or fMRI. Moreover, previous studies using EEG never employed out-of-the-lab protocols to explore this brain-behavioural link.

The fragrances perceived and rated as “invigorating” seem to lead to lower but longer brain activations. It also seems that the detergent on wet cloth is associated more to patterns of brain activity as shown by more correlations between the wet detergent scores and the AIDE parameters. Several associations are also found between the fragrance in the liquid detergent and the brain activation parameters, and almost none are found when the detergent is applied on dry cloth. The idea that fragrances perceived as energising can drive stronger brain activity is in line with other studies [51]. For the cloth assessment, it is worth noting how there is, to the best of our knowledge, no previous example in the literature of studies analysing brain activity in response to sensorial evaluation of different fabric materials at different levels of dampness. Previous research analysed the interaction of tactile and olfactive stimuli but focusing on different features like smoothness/roughness of the tactile sensation [52,53], hedonic experience during multisensory stimulation [1] or brain activity in the evaluation of positive vs. negative tactile and olfactive stimuli [54]. Our interpretation is that the wet feature of the cloth could generate a more complex cross-stimulation of the participant, therefore creating stronger brain responses when evaluating them compared to a dry cloth or the liquid detergent on its own.

## 5. Conclusion

This study provides a novel approach of fNIRS within the context of applied consumer research, with the results suggesting a reduction of cognitive load on repetitive household tasks (i.e. washing and folding laundry) following the introduction of fragrance.

The results of this study highlight two important points with regards to the efficacy of using fNIRS. Firstly, fNIRS seems to be a suitable tool to study brain activity in response to fragrances in naturalistic settings; and secondly, fNIRS is able to highlight subtle differences between Fragranced and Unfragranced conditions in naturalistic settings.

### Limitations & future work

Despite the novelty of the method and the uniqueness of this protocol in the fragrance industry, there are some limitations that should be considered when looking at the overall results of the study.

Naturalistic neuroimaging experiments pose unique challenges that can significantly undermine statistical power. These challenges mainly arise from the complex, dynamic nature of ecological experimental designs, which often introduce increased signal variability—including both the effects of interest and noise. For example, ambiguity in timing when an event occurs may prevent fully capturing neural and hemodynamic responses. Furthermore, the prolonged duration of events typical in realistic settings can cause increased interference from physiological noises that evolve slowly, such as respiration and infra-slow oscillations.

fNIRS signals can be strongly affected by changes in physiological responses. Motion artefacts and systemic cardiovascular adjustments were notably problematic in our study as participants moved freely around the space, leading to posture related changes in blood pressure and other physiological processes such as heart rate. This was also evident in the static phase of the experiment where participants are asked to smell fragrances repeatedly. When conducting similar experiments, it is recommended to measure physiological signals alongside fNIRS (i.e. systemic physiology augmented fNIRS) as additional biometric markers of outcome as well as to modulate the quality of the signal from fNIRS [55], and/or include short-separation in the array to minimise the impact of scalp and systemic interferences [56,25].

It is also important to note the challenges associated with olfactory experiments using fNIRS, as the main brain structures involved in olfaction are located in deep brain regions.

Variability introduced by individual differences in task engagement and brain responses to stimuli further compounded these issues, likely resulting in statistical analyses that did not withstand correction for multiple comparisons, especially with a modest sample size of 16 participants. To enhance statistical power in future research, increasing the number of subjects can help mitigate the additional variability in neural and behavioural responses.

Other hardware-based solutions can also be highly beneficial to account for systemic interferences, especially in naturalistic experiments. For instance, time domain NIRS (TD-NIRS) is more powerful than the classical continuous wave NIRS (CW) as it is able to separate the superficial contribution from the underlying deeper brain signals, giving a more accurate recovery of the brain hemodynamic responses [57]. High density diffuse optical tomography (HD-DOT) systems are also superior to typical sparse CW devices as, even if also based on the CW technology, they provide measurements at several depths and a larger availability of short separation channels, hence improving the reduction of superficial contamination [20].

In this experiment, the timings of the events of interest were manually annotated by the experimenter; eye-tracking or video cameras were not recorded during the current study for logistical reasons. Future research should consider incorporating behavioural tracking tools such as eye-tracking, video cameras and motion-tracking to precisely identify events of interest. This would be beneficial in making the identification of salient events less time consuming and more automatic [58], and in conjunction with machine learning and AI approaches [6]. Supplementing these with physiological measurements (e.g., heart rate, respiration) not only aids in accounting for physiological noise contaminating the fNIRS signals but also provides additional data for investigating experimental effects.

Adopting a Bayesian statistical framework could be particularly beneficial in this context. Through the specification of priors, researchers can readily incorporate existing knowledge to constrain the identification of events of interest and more effectively distinguish signal from noise [58]. Bayesian approaches enable formal model comparison that is well-suited to handle the trade-off between model complexity and accuracy, helping to identify the best model that explains the data while avoiding overfitting or overgeneralization [59]. Furthermore, Bayesian model comparison can be used to formally test for evidence of an effect and compare it to the evidence for the null hypothesis, eliminating the need for corrections for multiple comparisons, which are often at risk of being overly conservative [60].

Future research should also consider whether the findings of this research transcend to other consumer relevant tasks where fragrance would be an appropriate test stimulus and whether there are sex differences in fragrance perception both in the context of household activities and more broadly. Likewise, when considering alternative applications from these findings, additional research into the use of fNIRS in naturalistic settings could help promote the use of fNIRS capabilities where alternative techniques may be unsuitable.

## Supporting information

10.6084/m9.figshare.26381053 Supplement 1 Table with data, additional results figures https://doi.org/10.6084/m9.figshare.26381053

## Data Availability

Data underlying the results presented in this paper are available in the supplementary materials.

## Supplemental document

See Supplement 1 for supporting content.
