# Supplementary material for: Naturalistic approach to investigate the neural correlates of a laundry cycle with and without fragrance
Source: Biomed Opt Express. 2024 Aug 23;15(9):5461–78. doi: 10.1364/BOE.528275 (PMC11407240; doi:10.1364/BOE.528275)
Supplement: Supplement 1 — https://doi.org/10.6084/m9.figshare.26381053 [file boe-15-9-5461-s001.pdf]

Supplemental Document

Biomedical Optics EXPRESS

Naturalistic approach to investigate the neural correlates of a laundry cycle with and without fragrance: supplement

GIULIANO GAETA,[1] NATALIE GUNASEKARA,[2,*] PAOLA PINTI,[2,3] ANDREW LEVY,[3] EMILIA PARKKINEN,[1] EMILY KONTARIS,[1] AND ILIAS TACHTSIDIS[2,3]

[1] *Health and Well-being Centre of Excellence, Givaudan UK Limited, Ashford, UK*
[2] *Department of Medical Physics and Biomedical Engineering, University College London, London, UK*
[3] *Metabolight Ltd, Croydon, UK*
[*] *natalie.gunasekara.20@ucl.ac.uk*

This supplement published with Optica Publishing Group on 23 August 2024 by The Authors under the terms of the Creative Commons Attribution 4.0 License in the format provided by the authors and unedited. Further distribution of this work must maintain attribution to the author(s) and the published article's title, journal citation, and DOI.

Supplement DOI: https://doi.org/10.6084/m9.figshare.26381053

Parent Article DOI: https://doi.org/10.1364/BOE.528275

Supplementary Materials

Table S1

Table 1 – Anatomical locations of the fNIRS channels

Ch. number	MNI coordinates x	y	z	BA-anatomy	Probability
1	-47	45	24	45 - pars triangularis Broca's area<br>46 - Dorsolateral prefrontal cortex	0.75875<br>0.24125
2	-30	45	43	9 - Dorsolateral prefrontal cortex	0.87615
3	-49	50	-1	46 - Dorsolateral prefrontal cortex	0.91111
4	-34	64	-9	10 - Frontopolar area<br>11 - Orbitofrontal area<br>47 - Inferior prefrontal gyrus	0.25573<br>0.46947<br>0.20229
6	-25	69	4	10 - Frontopolar area<br>11 - Orbitofrontal area	0.62542<br>0.37458
7	-14	68	24	10 - Frontopolar area	1
8	-11	46	52	8 - Includes Frontal eye fields<br>9 - Dorsolateral prefrontal cortex	0.2249<br>0.7751
9	2	55	40	9 - Dorsolateral prefrontal cortex	0.90871
10	13	47	52	8 - Includes Frontal eye fields<br>9 - Dorsolateral prefrontal cortex	0.22358<br>0.77642
11	-12	73	-4	10 - Frontopolar area<br>11 - Orbitofrontal area	0.44444<br>0.55556
12	3	69	13	10 - Frontopolar area	1
13	15	73	-5	10 - Frontopolar area<br>11 - Orbitofrontal area	0.41667<br>0.58333
14	17	68	25	10 - Frontopolar area	1
15	29	69	4	10 - Frontopolar area<br>11 - Orbitofrontal area	0.65552<br>0.34448
16	45	55	16	46 - Dorsolateral prefrontal cortex	0.852
17	34	45	43	9 - Dorsolateral prefrontal cortex	0.85714
18	50	43	26	45 - pars triangularis Broca's area	0.8937
19	38	65	-9	10 - Frontopolar area<br>11 - Orbitofrontal area<br>47 - Inferior prefrontal gyrus	0.24901<br>0.40316<br>0.26482
20	51	50	1	46 - Dorsolateral prefrontal cortex	0.84859

Figure S1

Figure S1. Depicts the activation onsets for washing, hanging and folding for all participants and all channels

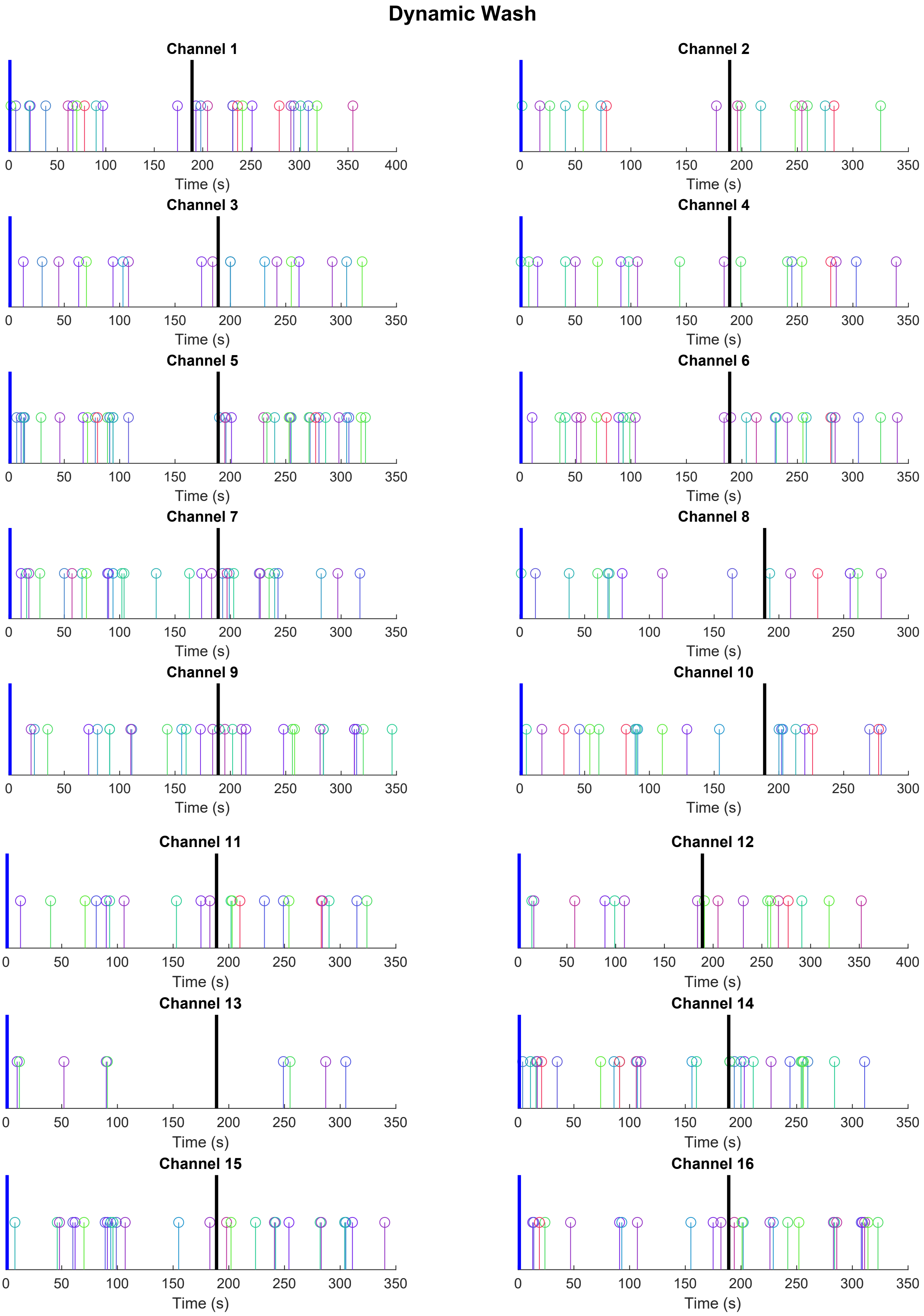

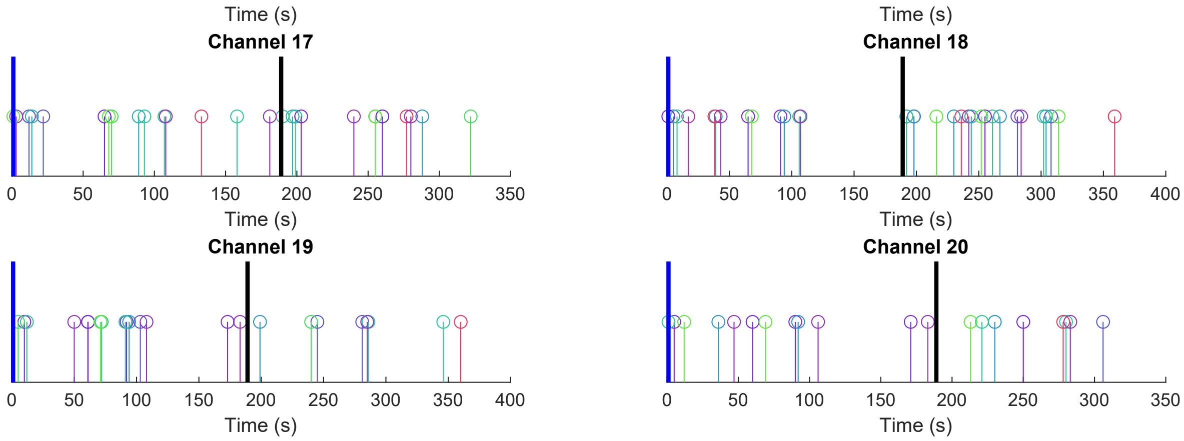

Dynamic Hang

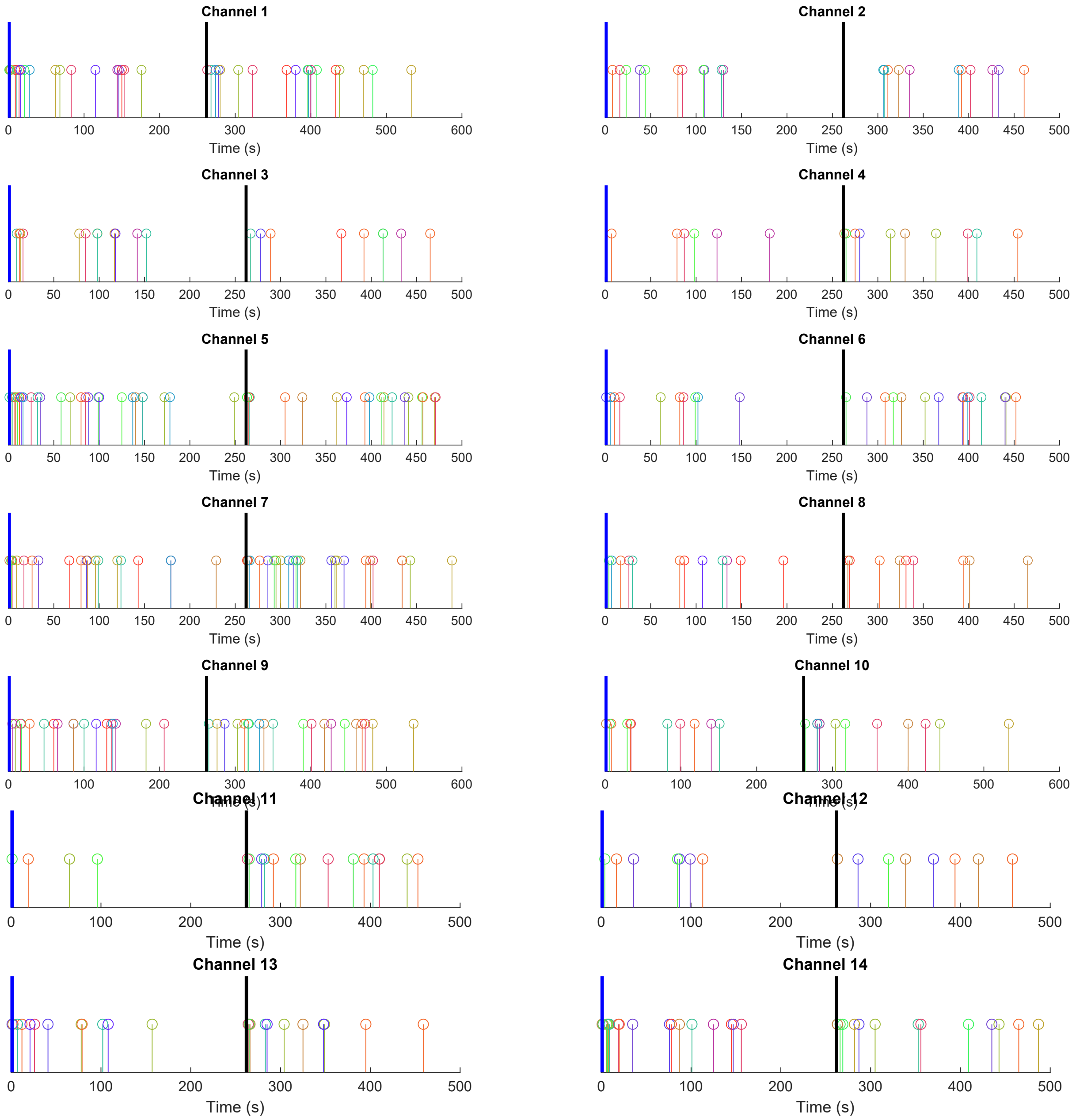

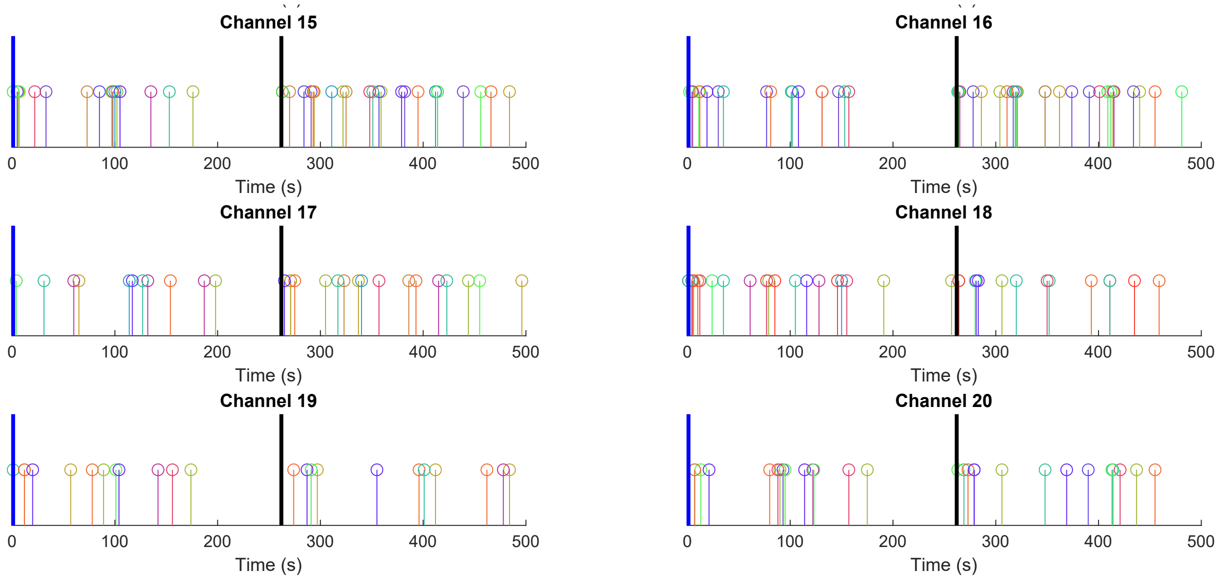

Dynamic Fold

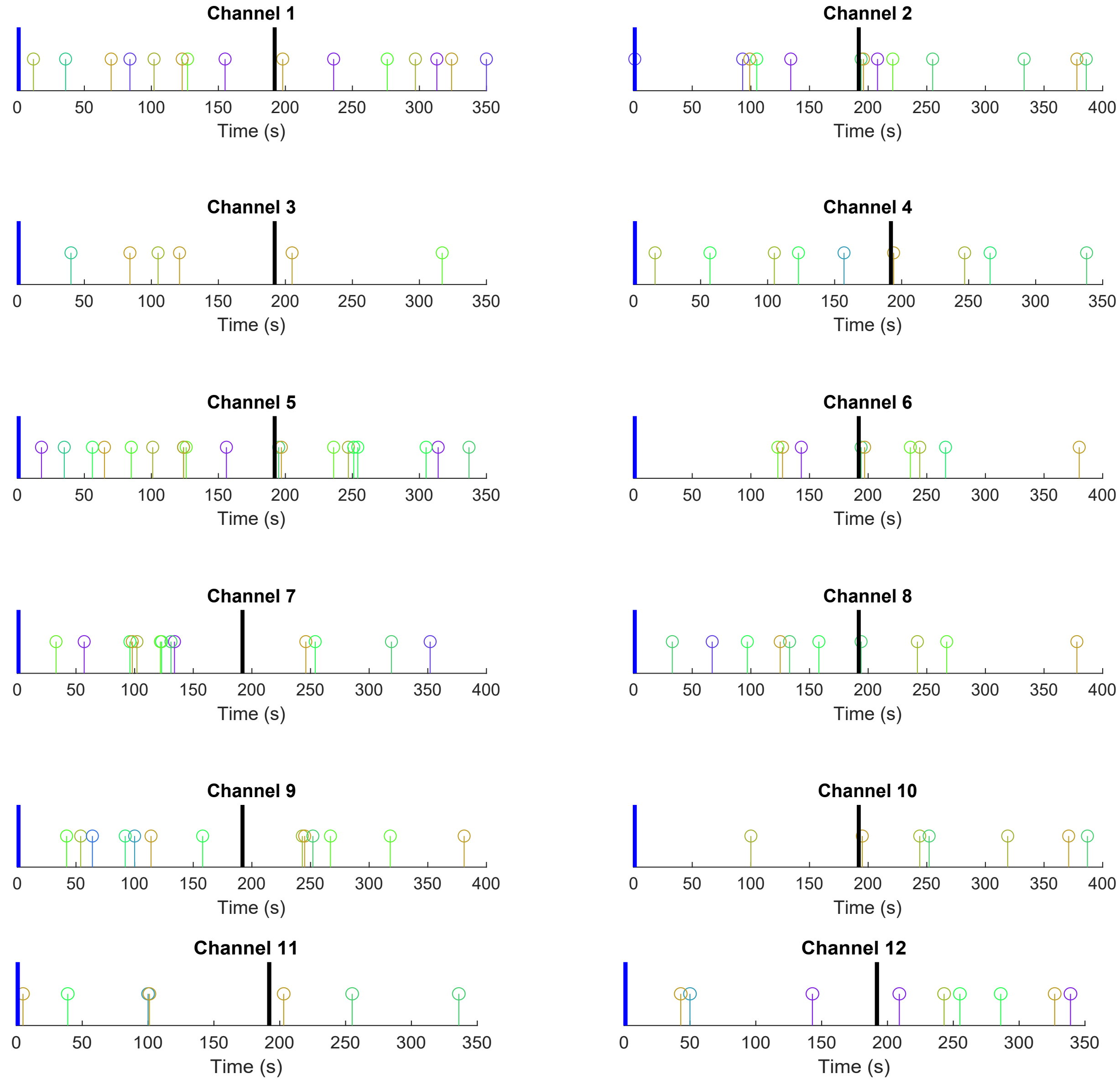

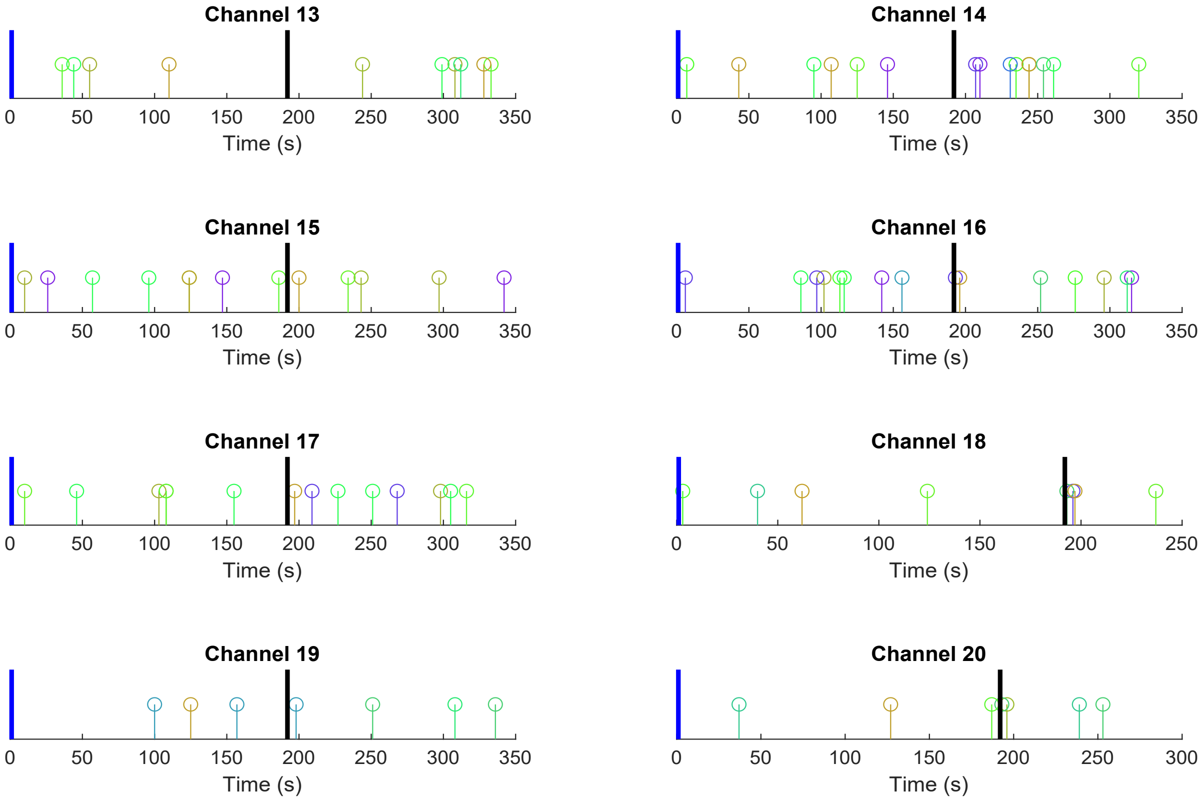
Channel 13
0 50 100 150 200 250 300 350
Time (s)
Channel 14
0 50 100 150 200 250 300 350
Time (s)
Channel 15
0 50 100 150 200 250 300 350
Time (s)
Channel 16
0 50 100 150 200 250 300 350
Time (s)
Channel 17
0 50 100 150 200 250 300 350
Time (s)
Channel 18
0 50 100 150 200 250
Time (s)
Channel 19
0 50 100 150 200 250 300 350
Time (s)
Channel 20
0 50 100 150 200 250 300
Time (s)

.

Table S2

Table S2. Depicts the means and standard deviations for the data used in statistical tests, separated by action type (wash, hang, fold, static) and detergent type.

**Dynamic Wash Fragranced**	Max t value		Mean duration		Mean t value		onset number	
Channel	Mean	Std Dev	Mean	Std Dev	Mean	Std Dev	Mean	Std Dev
Ch 01	8.29	1.38	16.60	7.85	7.86	1.13	1.50	0.76
Ch 02	7.02	1.00	17.71	6.05	7.00	1.01	1.14	0.38
Ch 03	8.35	1.95	13.06	1.99	7.79	1.92	1.67	1.03
Ch 04	7.65	2.31	17.03	4.43	6.95	1.52	1.83	0.75
Ch 05	7.51	2.08	13.71	7.35	6.91	1.68	1.50	0.52
Ch 06	7.39	1.83	13.63	7.07	7.01	1.45	1.63	0.74
Ch 07	6.82	1.26	17.64	6.72	6.59	0.95	1.42	0.67
Ch 08	7.45	2.26	9.43	9.64	7.19	2.02	1.29	0.49
Ch 09	7.62	1.30	16.83	8.64	6.86	1.19	1.75	0.71
Ch 10	6.93	2.31	14.39	7.19	6.37	1.31	1.44	0.53
Ch 11	7.84	2.16	15.97	8.99	7.11	1.49	1.67	0.82
Ch 12	6.82	1.73	19.33	8.23	6.28	0.82	1.75	0.96
Ch 13	6.90	3.00	9.83	7.32	6.15	1.76	1.67	0.58
Ch 14	7.09	1.60	12.92	10.72	6.74	1.53	1.45	0.69
Ch 15	7.23	2.10	17.52	4.24	6.94	1.77	1.56	0.73
Ch 16	9.82	2.78	14.25	6.33	8.97	1.44	1.83	0.98
Ch 17	6.49	1.40	16.23	6.25	6.20	1.07	1.50	0.71
Ch 18	8.56	2.72	14.83	8.94	7.65	1.49	1.44	0.53
Ch 19	7.89	2.33	18.09	6.81	7.13	1.82	1.67	0.87
Ch 20	7.81	2.77	20.74	14.53	6.78	1.28	1.71	0.95
**Dynamic Wash Un-fragranced**	Max t value		Mean duration		Mean t value		onset number	
Channel	Mean	Std Dev	Mean	Std Dev	Mean	Std Dev	Mean	Std Dev
Ch 01	7.44	1.83	18.40	7.64	7.20	1.84	1.60	0.52
Ch 02	7.19	1.91	16.90	10.29	6.48	1.32	1.80	0.84
Ch 03	8.63	3.47	16.33	9.71	8.05	3.64	1.50	0.55
Ch 04	7.85	1.43	10.30	8.63	7.18	1.09	1.60	0.55

Ch 05	7.49	1.77	16.16	7.57	7.18	1.50	1.25	0.58
Ch 06	8.44	2.49	11.53	6.27	7.83	2.28	1.56	1.01
Ch 07	6.29	1.10	16.85	6.85	6.18	1.05	1.20	0.42
Ch 08	7.00	0.86	18.25	2.82	6.95	0.92	1.17	0.41
Ch 09	6.65	1.36	15.73	8.46	6.45	1.25	1.55	0.69
Ch 10	6.94	2.07	9.43	8.96	6.78	2.11	1.29	0.49
Ch 11	7.73	1.66	15.06	8.98	7.35	1.69	1.83	0.75
Ch 12	7.25	3.08	16.50	11.13	6.68	2.02	1.67	1.03
Ch 13	7.14	0.55	14.50	10.11	6.65	0.30	1.33	0.58
Ch 14	6.73	1.51	15.25	5.88	6.38	1.16	1.40	0.52
Ch 15	8.51	2.59	17.17	8.16	7.89	2.11	1.56	0.73
Ch 16	9.76	4.28	17.17	9.55	8.43	2.42	1.50	0.71
Ch 17	6.99	1.58	18.39	8.25	6.67	1.41	1.44	0.53
Ch 18	7.79	2.57	16.82	8.80	7.36	2.60	1.82	0.75
Ch 19	6.88	1.19	17.71	6.32	6.66	0.90	1.14	0.38
Ch 20	6.89	1.41	13.79	6.04	6.80	1.26	1.14	0.38

**Dynamic Hang Fragranced**	Max t value		Mean duration		Mean t value		onset number	
Channel	Mean	Std Dev	Mean	Std Dev	Mean	Std Dev	Mean	Std Dev
Ch 01	7.52	2.40	17.14	11.96	6.75	1.27	1.64	0.81
Ch 02	8.06	2.69	12.00	8.44	6.86	1.31	1.83	0.75
Ch 03	7.14	1.62	11.34	8.64	6.64	1.27	1.63	1.06
Ch 04	6.22	0.76	19.50	4.80	5.94	0.40	1.50	0.58
Ch 05	8.03	2.10	12.90	5.40	7.25	1.64	2.00	0.96
Ch 06	6.92	1.78	12.79	8.49	6.37	1.15	1.43	0.53
Ch 07	7.82	2.95	9.46	6.51	6.71	1.45	1.67	0.89
Ch 08	6.75	1.36	13.58	5.57	6.40	1.07	1.50	0.76
Ch 09	7.28	2.12	10.83	7.93	6.64	1.51	1.64	0.81
Ch 10	6.63	0.97	9.62	5.15	6.48	1.07	1.57	0.79
Ch 11	6.73	2.31	18.75	5.25	6.73	2.31	1.00	0.00
Ch 12	7.09	1.59	16.40	10.57	6.80	1.55	1.40	0.55

Ch 13	6.39	1.14	20.07	12.58	6.02	0.80	1.71	0.49
Ch 14	7.29	2.10	12.69	6.73	7.08	2.03	1.38	0.51
Ch 15	7.01	1.95	14.79	6.05	6.62	1.50	1.42	0.51
Ch 16	7.12	1.56	13.24	6.87	6.61	0.88	1.64	0.81
Ch 17	6.10	1.18	16.46	8.74	5.86	0.72	1.38	0.74
Ch 18	7.49	2.19	13.58	7.42	6.88	1.82	1.91	1.14
Ch 19	7.63	0.83	12.06	6.13	7.36	0.72	1.50	0.53
Ch 20	7.73	2.31	14.22	7.04	6.85	1.33	1.56	0.53
**Dynamic Hang Unfragranced**	Max t value		Mean duration		Mean t value		onset num	
Channel	Mean	Std Dev	Mean	Std Dev	Mean	Std Dev	Mean	Std Dev
Ch 01	6.90	1.37	20.30	8.08	6.23	0.88	1.73	0.79
Ch 02	8.01	2.59	17.69	6.43	7.34	1.81	1.57	0.79
Ch 03	7.59	2.25	11.90	5.48	7.22	1.82	1.29	0.76
Ch 04	6.67	1.72	19.86	7.41	6.08	0.95	1.43	0.53
Ch 05	7.01	2.43	14.52	8.46	6.48	1.12	1.36	0.74
Ch 06	7.06	1.07	11.57	6.31	6.80	0.99	1.50	0.85
Ch 07	7.76	2.57	10.41	5.47	6.72	1.46	2.00	1.22
Ch 08	6.71	0.25	13.50	10.38	6.16	0.38	2.25	1.26
Ch 09	6.80	1.83	8.48	4.99	6.10	1.21	1.82	0.87
Ch 10	6.57	1.28	12.39	5.63	6.11	0.93	1.33	0.50
Ch 11	6.71	1.51	17.60	6.58	6.06	0.79	1.88	0.83
Ch 12	7.66	2.03	8.17	4.56	6.48	0.95	2.00	0.82
Ch 13	8.06	2.12	21.21	6.94	7.27	1.10	1.71	0.49
Ch 14	7.79	2.61	10.95	8.11	7.34	2.56	1.40	0.52
Ch 15	7.44	1.75	12.08	6.81	6.83	1.37	2.08	0.64
Ch 16	7.62	2.14	14.67	6.81	6.68	1.19	2.00	0.91
Ch 17	6.59	1.68	9.80	5.57	6.05	0.77	1.70	0.82
Ch 18	7.18	2.22	16.44	10.99	6.64	1.74	1.56	0.53
Ch 19	7.18	1.51	16.14	8.55	6.46	0.95	1.83	0.98
Ch 20	7.10	1.45	14.17	10.20	6.73	1.43	1.56	0.53

**Dynamic Fold Fragranced**	Max t value		Mean duration		Mean t value		onset number	
Channel	Mean	Std Dev	Mean	Std Dev	Mean	Std Dev	Mean	Std Dev
Ch 01	6.30	1.41	18.88	10.55	6.30	1.41	1.00	0.00
Ch 02	8.27	2.75	19.00	5.35	7.71	1.90	1.25	0.50
Ch 03	7.09	1.91	16.25	5.19	7.09	1.91	1.00	0.00
Ch 04	7.19	0.32	16.00	3.50	6.50	0.82	1.67	0.58
Ch 05	6.80	1.72	15.00	6.72	6.49	1.35	1.25	0.46
Ch 06	7.13	2.74	9.00	8.54	7.13	2.74	1.00	0.00
Ch 07	6.19	0.80	16.57	6.35	6.05	0.72	1.29	0.49
Ch 08	6.48	1.35	11.00	8.84	6.24	1.14	1.50	0.58
Ch 09	5.46	0.62	9.86	10.16	5.46	0.62	1.00	0.00
Ch 10	6.70	0.00	16.00	0.00	6.70	0.00	1.00	0.00
Ch 11	5.98	1.78	16.33	10.12	5.53	1.00	1.33	0.58
Ch 12	5.39	0.59	20.00	3.61	5.39	0.59	1.00	0.00
Ch 13	5.70	0.66	11.00	8.49	5.70	0.66	1.00	0.00
Ch 14	6.19	0.92	8.60	7.16	6.16	0.95	1.20	0.45
Ch 15	5.67	0.91	9.64	6.14	5.62	0.91	1.14	0.38
Ch 16	6.57	1.32	16.79	6.77	6.48	1.38	1.14	0.38
Ch 17	6.26	1.34	10.50	6.38	5.49	0.49	2.00	1.00
Ch 18	8.38	3.22	16.00	7.12	8.38	3.22	1.00	0.00
Ch 19	8.62	4.31	12.00	11.31	7.17	2.26	1.50	0.71
Ch 20	7.10	0.43	9.00	8.00	7.10	0.43	1.00	0.00
**Dynamic Fold Unfragranced**	Max t value		Mean duration		Mean t value		onset number	
Channel	Mean	Std Dev	Mean	Std Dev	Mean	Std Dev	Mean	Std Dev
Ch 01	6.16	1.86	15.40	6.23	5.78	1.22	1.40	0.55
Ch 02	6.89	1.49	17.25	3.86	5.92	0.60	2.00	1.41
Ch 03	5.51	0.35	21.50	3.54	5.51	0.35	1.00	0.00
Ch 04	6.42	2.14	16.00	9.76	6.42	2.14	1.00	0.00
Ch 05	5.66	0.90	16.86	10.80	5.60	0.88	1.29	0.49
Ch 06	5.64	0.59	11.50	11.43	5.50	0.33	1.20	0.45

Ch 07	5.37	0.53	23.00	5.35	5.37	0.53	1.00	0.00
Ch 08	5.64	0.42	9.50	7.14	5.64	0.42	1.00	0.00
Ch 09	5.37	0.44	11.50	8.44	5.36	0.45	1.20	0.45
Ch 10	6.03	1.09	11.75	12.53	5.79	0.74	1.50	0.58
Ch 11	8.26	3.46	15.75	11.67	8.21	3.54	1.50	0.71
Ch 12	5.76	0.55	7.88	3.71	5.66	0.61	1.50	0.58
Ch 13	6.31	1.19	8.83	8.21	6.31	1.19	1.00	0.00
Ch 14	5.28	0.56	9.78	8.54	5.28	0.56	1.00	0.00
Ch 15	6.23	1.17	17.00	10.68	6.23	1.17	1.00	0.00
Ch 16	5.62	0.51	19.14	10.56	5.62	0.51	1.00	0.00
Ch 17	5.64	0.59	24.17	11.77	5.52	0.54	1.33	0.52
Ch 18	5.39	0.35	21.25	8.66	5.39	0.35	1.00	0.00
Ch 19	7.16	2.40	20.83	3.55	7.15	2.41	1.33	0.58
Ch 20	5.28	0.31	19.17	5.97	5.19	0.30	1.33	0.58

**Static Dry Fragranced**	Max t value		Mean duration		Mean t value		onset number	
Channel	Mean	Std Dev	Mean	Std Dev	Mean	Std Dev	Mean	Std Dev
Ch 01	6.48	1.38	13.86	6.18	6.38	1.32	1.14	0.38
Ch 02	6.24	0.47	18.00	14.53	6.24	0.47	1.00	0.00
Ch 03	5.94	0.93	21.00	7.87	5.94	0.93	1.00	0.00
Ch 04	7.29	2.08	17.17	7.86	7.29	2.08	1.00	0.00
Ch 05	6.38	0.97	18.30	8.14	6.24	0.87	1.10	0.32
Ch 06	6.25	1.01	14.25	8.30	6.18	1.06	1.25	0.50
Ch 07	6.43	1.37	18.38	9.36	6.43	1.37	1.00	0.00
Ch 08	7.26	0.47	11.00	10.15	7.26	0.47	1.00	0.00
Ch 09	6.13	0.89	20.08	10.77	6.13	0.89	1.00	0.00
Ch 10	7.54	1.35	22.67	3.39	7.45	1.34	1.17	0.41
Ch 11	7.12	1.46	26.67	8.50	7.12	1.46	1.00	0.00

Ch 12	6.42	0.88	19.50	12.02	6.42	0.88	1.00	0.00
Ch 13	6.64	0.84	5.00	6.24	6.64	0.84	1.00	0.00
Ch 14	6.76	1.39	18.00	4.69	6.66	1.41	1.13	0.35
Ch 15	6.66	1.80	13.00	4.24	6.34	1.40	1.25	0.50
Ch 16	5.79	0.97	19.00	7.71	5.79	0.97	1.00	0.00
Ch 17	7.79	2.06	16.00	5.59	7.79	2.06	1.00	0.00
Ch 18	5.78	1.05	20.25	13.52	5.78	1.05	1.00	0.00
Ch 19	6.66	1.09	16.75	8.66	6.35	0.89	1.33	0.52
Ch 20	6.64	0.74	19.17	7.55	6.64	0.74	1.00	0.00

**Static Dry Unfragranced**	Max t value		Mean duration		Mean t value		onset number	
Channel	Mean	Std Dev	Mean	Std Dev	Mean	Std Dev	Mean	Std Dev
Ch 01	7.00	1.47	15.50	6.35	6.93	1.54	1.17	0.41
Ch 02	6.50	1.35	17.00	4.69	6.50	1.35	1.00	0.00
Ch 03	5.98	1.30	20.50	10.88	5.89	1.32	1.17	0.41
Ch 04	6.44	2.13	14.00	8.16	6.40	2.15	1.13	0.35
Ch 05	6.45	1.42	18.67	5.98	6.45	1.43	1.11	0.33
Ch 06	6.24	0.00	16.00	0.00	6.24	0.00	1.00	0.00
Ch 07	6.66	1.63	22.30	6.34	6.66	1.63	1.00	0.00
Ch 08	5.59	0.45	20.67	9.07	5.59	0.45	1.00	0.00
Ch 09	6.87	1.33	25.50	4.68	6.87	1.33	1.00	0.00
Ch 10	7.48	2.11	24.67	2.89	7.48	2.11	1.00	0.00
Ch 11	6.74	2.55	18.50	17.68	5.80	1.22	1.50	0.71
Ch 12	6.05	0.51	22.20	8.58	6.05	0.51	1.00	0.00
Ch 13	6.64	1.18	15.00	11.95	6.35	0.57	1.17	0.41

Ch 14	7.25	1.22	21.78	11.29	7.18	1.27	1.11	0.33
Ch 15	6.27	1.22	19.75	11.40	6.27	1.22	1.00	0.00
Ch 16	6.40	1.73	27.43	9.66	6.23	1.64	1.14	0.38
Ch 17	6.20	1.54	18.83	10.93	6.20	1.54	1.00	0.00
Ch 18	6.09	0.94	19.33	13.87	6.09	0.94	1.00	0.00
Ch 19	7.12	1.31	18.75	6.78	7.06	1.36	1.17	0.41
Ch 20	6.15	1.54	21.00	13.02	6.15	1.54	1.00	0.00

**Static Wet Fragranced**	Max t value		Mean duration		Mean t value		onset number	
Channel	Mean	Std Dev	Mean	Std Dev	Mean	Std Dev	Mean	Std Dev
Ch 01	7.53	2.56	17.50	7.33	7.53	2.56	1.00	0.00
Ch 02	7.63	0.10	30.00	19.80	7.63	0.10	1.00	0.00
Ch 03	6.91	1.61	19.75	12.04	6.91	1.61	1.00	0.00
Ch 04	6.49	1.55	12.50	11.09	6.40	1.53	1.25	0.50
Ch 05	6.28	1.33	22.83	6.34	6.28	1.33	1.00	0.00
Ch 06	5.69	0.00	15.00	0.00	5.69	0.00	1.00	0.00
Ch 07	5.92	1.13	16.25	5.25	5.92	1.13	1.00	0.00
Ch 08	8.48	3.95	21.67	6.03	8.48	3.95	1.00	0.00
Ch 09	6.93	2.17	20.00	8.27	6.93	2.17	1.00	0.00
Ch 10	6.23	0.85	18.44	7.88	6.15	0.83	1.13	0.35
Ch 11	8.96	2.65	19.00	16.09	8.96	2.65	1.00	0.00
Ch 12	5.99	1.08	19.50	6.81	5.99	1.08	1.00	0.00
Ch 13	5.69	0.49	16.33	1.53	5.69	0.49	1.00	0.00
Ch 14	6.66	1.93	18.63	8.69	6.59	1.90	1.08	0.29
Ch 15	6.26	1.15	13.20	7.05	6.26	1.15	1.00	0.00
Ch 16	7.66	2.03	17.25	6.18	7.66	2.03	1.00	0.00

Ch 17	6.88	1.99	19.00	11.40	6.88	1.99	1.00	0.00
Ch 18	6.58	1.29	21.80	10.31	6.58	1.29	1.00	0.00
Ch 19	6.55	1.79	18.00	10.93	6.38	1.80	1.20	0.45
Ch 20	8.00	0.50	21.00	15.52	7.60	0.98	1.33	0.58

**Static Wet Unfragranced**	Max t value		Mean duration		Mean t value		onset number	
Channel	Mean	Std Dev	Mean	Std Dev	Mean	Std Dev	Mean	Std Dev
Ch 01	6.29	0.07	24.50	12.02	6.29	0.07	1.00	0.00
Ch 02	6.10	1.36	22.33	13.65	6.10	1.36	1.00	0.00
Ch 03	6.57	1.84	23.00	9.87	6.57	1.84	1.00	0.00
Ch 04	5.79	0.72	22.33	6.11	5.79	0.72	1.00	0.00
Ch 05	6.45	0.92	21.67	5.01	6.45	0.92	1.00	0.00
Ch 06	6.55	0.00	16.00	0.00	6.55	0.00	1.00	0.00
Ch 07	6.29	0.73	20.40	5.03	6.29	0.73	1.00	0.00
Ch 08	7.33	2.09	15.75	8.54	7.33	2.09	1.00	0.00
Ch 09	7.08	1.69	17.33	9.83	7.08	1.69	1.00	0.00
Ch 10	6.36	1.45	19.00	7.14	6.36	1.45	1.00	0.00
Ch 11	7.69	3.11	29.50	0.71	7.69	3.11	1.00	0.00
Ch 12	6.27	2.06	17.33	6.35	6.27	2.06	1.00	0.00
Ch 13	6.97	2.59	19.40	12.42	6.97	2.59	1.00	0.00
Ch 14	6.91	0.75	18.00	5.61	6.91	0.75	1.00	0.00
Ch 15	6.04	1.33	21.75	9.74	6.04	1.33	1.00	0.00
Ch 16	5.96	1.59	15.50	14.85	5.96	1.59	1.00	0.00
Ch 17	5.80	0.64	19.00	4.80	5.80	0.64	1.00	0.00
Ch 18	6.52	1.37	26.33	10.88	6.52	1.37	1.00	0.00
Ch 19	5.53	0.58	21.43	5.13	5.53	0.58	1.00	0.00

Ch 20	6.49	0.70	19.40	6.15	6.49	0.70	1.00	0.00
**Static Liquid Fragranced**	Max t value		Mean duration		Mean t value		onset number	
Channel	Mean	Std Dev	Mean	Std Dev	Mean	Std Dev	Mean	Std Dev
Ch 01	6.62	1.06	19.00	6.20	6.62	1.06	1.00	0.00
Ch 02	6.80	0.00	25.00	0.00	6.80	0.00	1.00	0.00
Ch 03	6.94	0.81	32.33	2.52	6.94	0.81	1.00	0.00
Ch 04	5.99	0.00	28.00	0.00	5.99	0.00	1.00	0.00
Ch 05	6.17	1.24	24.50	6.53	6.17	1.24	1.00	0.00
Ch 06	NaN	NaN	NaN	NaN	NaN	NaN	NaN	NaN
Ch 07	5.45	0.68	20.67	8.62	5.45	0.68	1.00	0.00
Ch 08	6.61	0.66	27.00	14.14	6.61	0.66	1.00	0.00
Ch 09	8.23	1.88	28.00	6.93	8.23	1.88	1.00	0.00
Ch 10	6.28	0.52	14.00	4.24	6.28	0.52	1.00	0.00
Ch 11	6.33	2.23	29.00	14.14	6.33	2.23	1.00	0.00
Ch 12	7.24	0.00	21.00	0.00	7.24	0.00	1.00	0.00
Ch 13	6.66	0.19	19.00	21.21	6.66	0.19	1.00	0.00
Ch 14	7.41	1.34	30.25	2.99	7.41	1.34	1.00	0.00
Ch 15	5.44	0.61	24.50	9.88	5.44	0.61	1.00	0.00
Ch 16	5.73	0.72	21.33	12.50	5.73	0.72	1.00	0.00
Ch 17	6.69	0.00	23.00	0.00	6.69	0.00	1.00	0.00
Ch 18	5.89	0.79	21.80	8.61	5.89	0.79	1.00	0.00
Ch 19	5.89	0.63	14.50	3.54	5.89	0.63	1.00	0.00
Ch 20	6.43	1.04	25.33	9.07	6.43	1.04	1.00	0.00
**Static Liquid Unfragranced**	Max t value		Mean duration		Mean t value		onset number	

Channel	Mean	Std Dev	Mean	Std Dev	Mean	Std Dev	Mean	Std Dev
Ch 01	8.21	0.70	14.00	2.83	8.21	0.70	1.00	0.00
Ch 02	5.68	0.65	22.00	14.14	5.68	0.65	1.00	0.00
Ch 03	5.82	0.00	27.00	0.00	5.82	0.00	1.00	0.00
Ch 04	6.22	1.35	22.33	7.51	6.22	1.35	1.00	0.00
Ch 05	6.21	1.08	18.60	12.14	6.21	1.08	1.00	0.00
Ch 06	6.06	0.93	20.33	2.08	6.06	0.93	1.00	0.00
Ch 07	5.88	0.97	24.00	4.24	5.88	0.97	1.00	0.00
Ch 08	6.35	0.66	17.50	3.54	6.35	0.66	1.00	0.00
Ch 09	6.93	2.10	19.00	6.04	6.93	2.10	1.00	0.00
Ch 10	6.89	2.10	22.60	8.08	6.89	2.10	1.00	0.00
Ch 11	6.20	0.91	26.50	0.71	6.20	0.91	1.00	0.00
Ch 12	7.24	2.83	17.33	9.02	7.24	2.83	1.00	0.00
Ch 13	7.39	3.40	21.00	1.41	7.39	3.40	1.00	0.00
Ch 14	6.10	0.57	25.50	7.85	6.10	0.57	1.00	0.00
Ch 15	5.79	0.96	26.75	4.79	5.79	0.96	1.00	0.00
Ch 16	7.08	1.08	15.00	5.66	7.08	1.08	1.00	0.00
Ch 17	6.88	1.33	17.25	1.71	6.88	1.33	1.00	0.00
Ch 18	6.76	1.58	18.75	6.40	6.74	1.59	1.25	0.50
Ch 19	5.94	0.60	23.00	1.41	5.94	0.60	1.00	0.00
Ch 20	5.90	0.46	15.00	7.07	5.90	0.46	1.00	0.00
